# Virtual Partnership Addressing Mental Health Crises: Mixed Methods Study of a Coresponder Program in Rural Law Enforcement

**DOI:** 10.2196/42610

**Published:** 2023-03-20

**Authors:** M Muska Nataliansyah, Kimberly A S Merchant, J Priyanka Vakkalanka, Luke Mack, Seth Parsons, Marcia M Ward

**Affiliations:** 1 Department of Surgery Collaborative for Healthcare Delivery Science Medical College of Wisconsin Milwaukee, WI United States; 2 Department of Health Management and Policy College of Public Health University of Iowa Iowa City, IA United States; 3 Department of Emergency Medicine University of Iowa Carver College of Medicine Iowa City, IA United States; 4 Avel eCare Sioux Falls, SD United States; 5 Department of Family Medicine University of South Dakota Sanford School of Medicine Sioux Falls, SD United States; 6 Department of Psychiatry University of South Dakota Sanford School of Medicine Sioux Falls, SD United States

**Keywords:** mental health, telehealth, rural health, coresponder model, implementation, mixed methods, community, technology, virtual care

## Abstract

**Background:**

A mental health crisis can create challenges for individuals, families, and communities. This multifaceted issue often involves different professionals from law enforcement and health care systems, which may lead to siloed and suboptimal care. The virtual crisis care (VCC) program was developed to provide rural law enforcement with access to behavioral health professionals and facilitated collaborative care via telehealth technology.

**Objective:**

This study was designed to evaluate the implementation and use of a VCC program from a telehealth hub for law enforcement in rural areas.

**Methods:**

This study used a mixed methods approach. The quantitative data came from the telehealth hub’s electronic record system. The qualitative data came from in-depth interviews with law enforcement in the 18 counties that adopted the VCC program.

**Results:**

Across the 181 VCC encounters, the telehealth hub's recommended disposition and the actual disposition were similar for remaining in place (n=141, 77.9%, and n=137, 75.7%, respectively), voluntary admission (n=9, 5.0%, and n=10, 5.5%, respectively), and involuntary committal (IVC; n=27, 14.9%, and n=19, 10.5%, respectively). Qualitative insights related to the VCC program's implementation, use, benefits, and challenges were identified, providing a comprehensive view of the virtual partnership between rural law enforcement and behavioral health professionals.

**Conclusions:**

Use of a VCC program likely averts unnecessary IVCs. Law enforcement interviews affirmed the positive impact of VCC due to its ease of use and the benefits it provides to the individuals in need, the first responders involved, law enforcement resources, and the community.

## Introduction

### Background

A mental health crisis can cause an emergency response, disruption, and long-term problems for individuals, families, and communities [1,2]. This multifaceted issue often involves law enforcement, health care, and justice systems, potentially leading to siloed and suboptimal care [3]. Enhancement in collaborative responses to and outcomes of mental health crises requires improving the care delivery model and organization of resources [4,5].

Various crisis response models have been developed to address the need for appropriate emergency responses for people with mental health conditions [6]. Among these models, 2 major examples are the crisis intervention team (CIT) model and the coresponder model [7]. The CIT model requires 40 hours of training to equip law enforcement officers with the needed knowledge and skills to coordinate mental health care. In contrast, the coresponder model emphasizes pairing law enforcement officers with behavioral health professionals [6,7]. Although law enforcement officers are often provided with some crisis intervention training, there are indications that working with behavioral health professionals can add layers of expertise and improve the service to communities [7,8]. Considering the potential value of partnership in addressing mental health crises, this study was designed to evaluate the implementation and use of a virtual crisis care (VCC) program from a telehealth hub for law enforcement in rural areas.

### Coresponder Model

Studies have shown that having behavioral health professionals collaborate with law enforcement officers on mental health crisis calls reduces the burden on the justice system and improves access to mental health care [9,10]. Most studies have been conducted on in-person mental health services in urban areas [11-14]. However, many rural areas are experiencing a workforce shortage of qualified behavioral health professionals, which makes the in-person coresponder model largely infeasible. An alternative is bringing a behavioral health professional virtually to a crisis encounter via telehealth to support law enforcement officers as they assist people in rural and underserved communities by offering timely behavioral health services.

One primary challenge in mental health emergencies encountered by law enforcement officers is the decision to commit people with mental health conditions involuntarily. This decision can be resource intensive and lead to suboptimal care [15]. A survey of involuntary admitted individuals showed that most of them felt pressured to be hospitalized and were more likely to report a subjective lack of improvement [16]. Studies also found that involuntary committal (IVC) is associated with poorer outcomes [17,18]. Legal procedures related to IVCs can vary by state and may include extensive requirements for documentation [19]. However, in most states, the IVC process requires a mental illness diagnosis and a judge’s signature to issue the IVC order [19].

Since geographical boundaries, local policies, and limitation of resources can shape the delivery of care, there is a need to explore the implementation of a coresponder model in rural areas [3,4,20]. This is especially relevant in South Dakota, where more than half of the population lives in rural areas and an estimated 10% of all emergency calls involve a person who may have a mental health crisis [21].

### Virtual Crisis Care Program

The Leona M. and Harry B. Helmsley Charitable Trust provided Avera eCARE (now known as Avel eCare) with a grant to establish and implement telehealth programs that would help meet the behavioral health needs of rural communities in South Dakota. Specifically, the grant supported the development of a mobile crisis care service for law enforcement and probation officers called VCC.

The VCC's pilot program was initiated in the field in January 2020. Over the subsequent first 9 months of the pilot program, the service was implemented in 18 county sheriff departments and 1 judicial circuit court probation district representing probation offices within 8 (44.4%) of those counties. Avera eCARE served as the hub for the VCC program and staffed it at all times with qualified behavioral health professionals who had experience in providing virtual and in-person consultations. Law enforcement officers in the participating rural counties were trained and supplied with electronic tablets to connect with the VCC hub for virtual mental health crisis support. The hub provided training materials and conducted sessions to help law enforcement officers get accustomed to the service. Law enforcement officers used VCC to acquire immediate and timely mental health evaluations by professionals trained in communicating and recognizing when someone would be a danger to themselves or others. The recommendations provided by the VCC connection informed the decisions made by law enforcement officers in caring for individuals in crisis.

The COVID-19 pandemic provided an opportunity to showcase the value of using telehealth to bridge this partnership. The use of familiar technology also contributed to the positive response and willingness to implement the innovative program. Overall, the virtual consultation aimed to de-escalate and stabilize the mental health condition of an individual during a crisis situation. After responding to a crisis call and conducting an initial assessment, law enforcement officers decided whether to offer and initiate the VCC service. Following a telehealth consultation with the person in crisis using the provided tablets, the VCC behavioral health professional shared their recommendation with law enforcement officers, who ultimately decided the disposition of the individual. Means restriction (ie, reducing a suicidal individual’s access to lethal means) and safety planning (ie, reducing the risk of harm to oneself) were strategies used to help individuals remain in place [22,23]. The hub team strived to collaboratively partner with local mental health community organizations to further assist the individuals establish outpatient resources, if needed.

## Methods

### Study Design

Using a mixed methods explanatory design, an independent team of researchers gathered data using both quantitative and qualitative approaches. The telehealth hub collected all quantitative data in its electronic record system. Variables assessed included the gender of the person served, mental health conditions identified at the time of the encounter, and the location of use. Specifically for location, *home* was identified when the incidence occurred at a residence, the *jail* was the county detention facility, and the *community* included any other public place or property. The recommended disposition by the telehealth hub and the actual disposition, which was verified by each county, were also captured and evaluated. These included remaining in place or getting admitted to a mental health facility (further classified as voluntary or involuntary). There may have also been continued law enforcement involvement until the individual was stabilized.

We tabulated several county-level measures to describe the rurality of these areas, health care coverage, and area deprivation. For rurality, we used the second-level rural-urban commuting area codes to classify as rural and also report the population density of these counties [24]. For health care access measures, we used data from the County Health Rankings data for these counties, including ratios of primary care providers and mental health providers [25]. Additionally, we described these counties by a measure of the area deprivation index percentile, comparing the rankings of these counties with national percentiles [26].

In-depth qualitative interviews were conducted to collect insights from users of the service. We used purposive sampling by inviting law enforcement officers from counties that adopted the VCC program to participate in a semistructured interview. These contacts were provided to the research team by the VCC hub team, indicating that the counties had experience using VCC and were willing to share perspectives. In the end, we were able to collect insights from the listed key informants who were most familiar with the VCC program from 10 different counties. This completion allowed us to be more confident in the generated findings, since it included the vast majority and full range of usage in the total sample. The telephone interviews—each ranging between 30 and 60 minutes—took place between January and May 2021. Two experienced qualitative researchers conducted the interviews. Both have years of experience conducting telehealth research and qualitative methods. Questions addressed topics including why their county was interested in VCC, the motivation to use the service, descriptions of typical and unique situations when used, community reactions and the willingness to use, and overall opinion of the service. We used these questions not only to gain insights into the implementation process but also to acquire some idea of how the community responded to the availability of VCC service.

The qualitative component included 14 interviews with 15 law enforcement officers in sheriff's departments in 10 counties, with 1 (10%) county extending interviews to city police department users. The interviews were audio-recorded, transcribed, and reviewed independently by 2 research team members. One coder had a background in health organization and implementation science, and the other was a qualitative specialist, having studied telehealth applications in multiple settings. They used an inductive qualitative analysis approach to identify relevant quotes and themes related to VCC use [27,28]. The researchers then met to discuss their findings, which led to the identification of themes and a composition of relevant quotes. The coders discussed the emerging findings with another research team member to enhance the validity of the results. Any discrepancies were discussed until consensus was reached. A final step was to classify the themes into domains and review all coding with the full research team. The process generated 3 domains that contained associated themes: (1) the *implementation of VCC* domain includes themes relevant to the initiation of the service, the need for the program, and why the program is important to law enforcement; (2) the *use of VCC* domain includes themes relevant to the experience of using the service or how law enforcement officers include the VCC program in their daily routine; and (3) the *benefits and challenges of VCC* domain includes themes relevant to the opinions of the service with lessons learned. The availability of multiple collaborators with different backgrounds and familiarity with the subject facilitated discussions with valuable perspectives that complemented each other. We used Microsoft Excel to manage and categorize quotes from different interviewees into themes and domains. The research followed the Consolidated Criteria for Reporting Qualitative Research (COREQ) for reporting purposes [29].

### Ethical Considerations

The project protocol was reviewed by the Institutional Review Board (IRB) of the University of Iowa and did not meet the regulatory definition of human subjects’ research, because the information sought from interviewees would not be private information concerning the interviewee—it would be information about the VCC services. All methods were carried out in accordance with relevant guidelines and regulations. Participation was voluntary, and no compensation was provided. Participants’ identities were confidential and de-identified, and data were presented anonymously.

## Results

### VCC Use in a Rural Setting

In total, 18 counties and 1 judicial circuit court probation district implemented VCC during the pilot program, accounting for 181 encounters during the study period (Table 1). Usage varied from 0 to 34 encounters per site and was unrelated to the length of time in use. About two-thirds of the consultations were with men (n=113, 62.4%), one-third with women (n=67, 37.0%), and 1 (0.6%) was missing this data. The most common location for using the service was in the community (n=81, 44.8%), followed by home (n=73, 40.3%), with a smaller percentage occurring in jail (n=27, 14.9%).

**Table 1:** Characteristics of individuals by VCC^a^ encounter setting.

Characteristics		Total (N=181), n (%)		Community (n=81, 44.8%), n (%)	Home (n=73, 40.3%), n (%)	Jail (n=27, 14.9%), n (%)
**Gender**						
	Male	113 (62.4)	43 (53.1)		46 (63.0)	24 (88.9)
	Female	67 (37.0)	37 (45.7)		27 (37.0)	3 (11.1)
	Missing	1 (0.6)	1 (1.2)		0	0
**Nature of request**						
	Suicidal ideation or self-harm	97 (53.6)	44 (54.3)		37 (50.7)	16 (59.3)
	Depression or anxiety	48 (26.5)	24 (29.6)		19 (26.0)	5 (18.5)
	Aggressive behavior	29 (16.0)	11 (13.6)		13 (17.8)	5 (18.5)
	Delusion or hallucinations	7 (3.9)	2 (2.5)		4 (5.5)	1 (3.7)

^a^VCC: virtual crisis care.

Encounters resulted in VCC consultations for various mental health situations, of which suicidal ideation or self-harm represented over half of all encounters (n=97, 53.6%). Other mental health situations identified for VCC consultations included depression or anxiety (n=48, 26.5%), aggressive or disruptive behavior or bullying (n=29, 16.0%), and delusion or hallucinations (n=7, 3.9%).

The 18 counties in this assessment have 78 census tracts included in them, of which 50 (64%) are considered rural, as defined by their rural-urban commuting area codes. The median population density (people per square mile) is 166 (IQR 8-1139). The average ratios for primary care providers and mental health providers per population are 1:1935 and 1:1004, respectively. Using the area deprivation index percentile comparing these counties to national estimates, the median percentile was 72 (IQR 58-82), indicating a relatively high level of socioeconomic disadvantage.

The behavioral health professional at the telehealth hub made recommendations directly to law enforcement officers at the conclusion of the VCC encounter (Table 2). The most common recommendation was that the person remain in place (n=141, 77.9%). The behavioral health professional occasionally recommended that the person in crisis be voluntarily admitted (n=9, 5.0%) or be involuntarily committed (n=27, 14.9%). Overall, the actual disposition agreed with the recommended disposition by VCC for 92% of encounters with a known disposition (ie, neither disposition was missing). The telehealth hub's recommended disposition and actual disposition were similar for remaining in place (n=141, 77.9%, and n=137, 75.7%, respectively) and voluntary admission (n=9, 5.0%, and n=10, 5.5%, respectively). In most encounters, the law enforcement officers followed the VCC providers’ recommendation but gave a rationale for the few instances when other actions were taken. Differences in recommended and actual dispositions for IVCs were primarily due to encounters in the community, such that only 8 (9.9%) of the community encounters resulted in an IVC compared to the recommended 14 (17.3%). Considering the limited sample, we only presented a descriptive statistic and did not conduct further statistical analyses to avoid misinterpretation of the results.

**Table 2:** Distribution of recommended and actual dispositions by location of incident.

Dispositions	Community (n=81, 44.8%), n (%)		Home (n=73, 40.3%), n (%)		Jail (n=27, 14.9%), n (%)		Total rec.^a^ (N=181), n (%)	Total act.^b^ (N=181), n (%)
	Rec.	Act.	Rec.	Act.	Rec.	Act.		
Remain in place	63 (77.8)	64 (79.0)	51 (69.9)	46 (63.0)	27 (100.0)	27 (100.0)	141 (77.9)	137 (75.7)
Voluntary admission^c^	2 (2.5)	3 (3.7)	7 (9.6)	7 (9.6)	0	0	9 (5.0)	10 (5.5)
IVC^d^	14 (17.3)	8 (9.9)	13 (17.8)	11 (15.1)	0	0	27 (14.9)	19 (10.5)
Law enforcement (other)^e^	0	4 (4.9)	0	1 (1.4)	0	0	0	5 (2.8)
Missing	2 (2.5)	2 (2.5)	2 (2.7)	8 (11.0)	0	0	4 (2.2)	10 (5.5)

^a^Rec: recommended disposition by the virtual crisis care (VCC) program.

^b^Act: actual disposition by law enforcement personnel.

^c^Admissions included higher levels of care in a health care setting, such as inpatient stay, emergency department visit, or transfer to a psychiatric facility.

^d^IVC: involuntary committal.

^e^Continuous monitoring by law enforcement until the individual was stabilized.

### Insights Into VCC Implementation in Rural Settings

In-depth interviews with 15 law enforcement officers from 10 counties provided helpful information about learning points from the VCC implementation in rural areas. These 10 counties experienced 155 (85.6%) of the 181 VCC encounters completed over the 18-month pilot.

Identified themes were grouped into 3 main categories: the initiation/implementation of VCC, the use of VCC, and the benefits and challenges of VCC (Table 3). Selected quotes representing the themes are presented in Table 4.

**Table 3:** Qualitative insights into the VCC^a^ program.

Themes and subthemes		Details
Theme 1: implementation of VCC		Demographic and geographic setting Resource-intensiveness of IVCs^b^ Relevant technology and service training
Theme 2: use of VCC		Presenting the service The consultation process Recommendations and decisions
Theme 3: benefits and challenges of VCC		
	Subtheme 3A: benefits	Mitigation of IVC procedures Better documentation for mental health crisis encounters Providing quality and efficient access to mental health experts
	Subtheme 3B: challenges	Technical concerns Limited network availability

^a^VCC: virtual crisis care.

^b^IVC: involuntary committal.

**Table 4:** Selected quotes on the VCC^a^ program in the rural United States.

Themes and subthemes		Quotes
**Theme 1: implementation of VCC**		
	Demographic and geographic setting	“We serve about 2350 square miles. We're a big county, but not a big population.” [Site C]
	Resource-intensiveness of IVCs^b^	“Usually, we burn about 24 man-hours every time we have a mental health commitment that's involuntary. And under state law, we're obligated. If we feel that a person is a harm to them self or to others, we're obligated to act. And so, it utilizes a lot of manpower and man-hours to accomplish that goal.” [Site I]
	Relevant technology and service training	“The majority of the training was how to use the tablets, how to log-in to those, how to make sure they're working, and then real simple instructions on going forward. We went through the criteria on what was needed for us to make the phone call, the questions we needed to ask of the person in crisis, the information we needed to gather before we made the phone call and got online with the doc.” [Site M]
**Theme 2: use of VCC**		
	Presenting the service	“So, if it's in the home, we'll talk to them about that we've got these options that we could take them in or we have a nice new program where they can talk with a behavioral health professional on an iPad within the comfort of their own home. And then that way somebody who is more trained than I am with mental health and counseling will have an opportunity to talk with them. If they agree to that, then we'll call the iPad, get that set up, call ahead for the online behavioral health professional, and inform them of the situation and the person's name.” [Site A]
	The consultation process	“If it's a person who I feel shouldn't be left alone, I stay in the room. But, normally, after gaining rapport with them and speaking with them, I tell them, “Hey, I'm going to give you some one-on-one time with the mental health professional. If you need anything, I'll be in the next room.” I feel like by me stepping out of the room and giving them some one-on-one time, it really opens them up to saying more to the mental health professional who's on the other end of the tablet.” [Site H]
	Recommendations and decisions	“And then when they're done, we would talk to the behavioral health professional and see what their recommendations are knowing that we're on scene and we still have the final say. But we'll take that into consideration.” [Site N]
**Theme 3A: benefits of VCC**		
	Potential mitigation of IVC procedures	“I think any of them that we've done the [VCC] on and follow the [mental health professional] recommendations to either turn to a safety plan or return to their home environment, I think all of those were prevented IVCs. I'm not saying they would've all been ordered for continued detention after the IVC, but I think every one of those 34 that the recommendation was to not continue with the IVC, which I think we've had two out of the 34 that they said, yeah, you should do an IVC anyway. So, 32 of them I think were prevented involuntary commitments.” [Site J]
	Better documentation for mental health crisis encounters	“It's documented very well. The whole encounter is very detailed in how it's logged. I think it's beneficial for us to get [the VCC report] back so we can write our report and be thorough.” [Site N]
	Providing quality and efficient access to mental health experts	“Using the [VCC] system has been fantastic both from the officer's point of view and from the client's point of view, the person who's in crisis. Because they tell us that. They don't get hauled away, taken away to a facility in another community where they're held until they're evaluated and deemed whether or not they can go home. In these cases [where VCC is used], they've been able to talk with somebody. A lot of times that seems to help, just being able to vent to the right person and then come up with some sort of game plan, a plan of action for them to follow up on whether it's seeing a behavioral health professional or whatever it may be.” [Site K]
**Theme 3B: challenges of VCC**		
	Technical concerns	“I know the one time with my juvenile, I called the number. I got a hold of the behavioral health professional. We set up an appointment via the iPad, but for some reason it wasn't connecting. I thought I had good cell reception. I don't know if it was some technical issue, but we just overcame that and she just talked to him on my cell phone.” [Site F]
	Limited network availability	“It happened one time where the internet was a little tough to deal with. And the only thing that happened was the subject and [mental health professional], they could hear each other, but as far as the screen and stuff, the screen would kind of pause. That has happened the one time, but everybody could hear everything. It was just you couldn't have that screen-to-screen interaction.” [Site B]

^a^VCC: virtual crisis care.

^b^IVC: involuntary committal.

#### The Implementation of VCC

The themes presented in this section highlight factors that influenced VCC implementation. The first factor that motivated VCC implementation was the demographic and geographic setting. The South Dakota county sheriff departments serve extensive rural areas with limited resources. Beyond the regular population of local counties, tourist activity can skyrocket the number of people and increase the workload for small county sheriff departments.

The second factor that motivated VCC implementation was the resource-intensiveness of IVCs. One important responsibility of county sheriff departments in South Dakota is to transport to mental health facilities individuals who are being involuntarily committed due to mental health crises. These transfers take time and money due to long travel distances—up to 500 miles in some cases; if the transfer is unnecessary, it creates undue strain on the person in crisis, the health system, and local law enforcement.

The third factor influencing VCC implementation was relevant training from the telehealth hub. As soon as county sheriff departments were on board to participate in this pilot VCC program, the VCC hub implementation staff provided equipment and training to the county law enforcement officers. The interviewees indicated the equipment was easy to use and simple to implement and that the training was straightforward. Test calls were encouraged, and ongoing training, troubleshooting, and communication were always available.

#### The Use of VCC

The themes in this section illustrate the process of using the VCC service. The first step in the consultation process was presenting the service to individuals with mental health conditions. Law enforcement officers responded to calls—most of them coming from family members or neighbors who had concerns for friends or loved ones—that presented requests ranging from welfare checks to crisis intervention. As shown in Table 1, more than half of the requests involved suicidal ideation or self-harm. Once law enforcement responded to a call of this nature, they then determined whether to use VCC based on their experience, comfort in offering, and the person in crisis.

The second step was to allow the individual in crisis time and space to consult with the VCC behavioral health professional, while maintaining safety for all. The telehealth encounter using the electronic tablet took between 15 and 60 minutes, depending on the circumstances. At the final step of the encounter, the behavioral health professional reconnected with law enforcement and provided a recommendation. Law enforcement officers had the option of carrying through with the VCC recommendation or choosing a different course of action based on additional knowledge or extenuating circumstances.

In this study, law enforcement overwhelmingly followed the VCC behavioral health professional's recommendation, but in the few instances when other actions were taken, they gave a rationale for the departure. These exceptions included hearing the person lie, intoxication, having the person attempt self-harm after the call, being called back to the home within hours, having the safety plan refused or not implemented after the call, or, in several situations, having to follow through with an arrest warrant.

#### The Benefits and Challenges of VCC

The themes in this section summarized lessons learned from the VCC pilot program and included insights into the benefits and challenges of the VCC service.

##### Benefits

According to law enforcement, the various benefits of the VCC program have shown a positive direct impact on individuals experiencing mental health crises and the law enforcement officers involved, plus the broader value of having the service in these rural communities.

The first benefit of VCC is potential mitigation of IVC procedures, which could lighten the use of already limited resources. South Dakota has a legal procedure that law enforcement must follow for IVC. This includes proper documentation, evaluations by a qualified mental health professional, and forms to be signed, followed by County Bureau of Mental Illness review and decision, which can be a lengthy process [30]. One interviewee shared that before a resource like VCC was available, just the mention or threat of suicide would prompt an IVC in past scenarios. Multiple interviewees emphasized how working with the VCC hub behavioral health professional facilitates an opportunity to acquire more information, promote crisis reduction, work out a safety plan, and frequently prevent an IVC from occurring.

The second benefit was better documentation for mental health crisis encounters. Postencounter reports from VCC behavioral health professionals shared with law enforcement provided a detailed, timed report about the encounter and interaction with the individual in crisis and the rationale for the disposition recommendation. This documentation has proven helpful to the sheriff departments, which attach the information to law enforcement incident reports, by developing a history and patterns for individuals that could be beneficial in the future, as verification for decisions to pursue IVCs when necessary and as documentation of actions supporting stay-in-place decisions.

A third benefit of VCC was providing the rural community with quality and efficient access to mental health experts. Quality elements of VCC, such as timeliness, convenience, and expertise in assessment, were noted as benefits. It was noted that the virtual conversation between the individual in crisis and the behavioral health professional often diffuses the situation. Officers commented that they greatly value the involvement of expertly trained behavioral health professionals. In addition, interviewees reported that VCC helps them do their jobs better for the communities they serve.

##### Challenges

As in any pilot program, the law enforcement participants understood VCC to be a work in progress. From their experience, interviewees provided suggestions for improvements to further meet law enforcement needs.

One of the challenges was presenting the VCC technology out in the field. When the VCC program was initialized, some officers experienced technical difficulties or connectivity issues. Another difficulty was encouraging an irrational person in crisis to interact with the telehealth tablet and engage the VCC behavioral health professional. However, as 1 officer stated, the timing worked in favor of VCC because COVID-19 has led to many people becoming more familiar with using virtual platforms for communication.

Another challenge was limited network availability in highly rural areas. To address this issue, law enforcement officers used an internet hotspot or cell phone booster technology in patrol cars and were aware of locations in the county where broadband service was available. In fact, when preparing to launch VCC in some counties, the officers did sweeps of their counties' geography to note good broadband locations. Consequently, some encounters moved from home to car or a nearby open space or building.

## Discussion

### Principal Findings

Law enforcement officers are commonly the first responders to a mental health crisis in rural communities. By partnering virtually with behavioral health professionals in these situations, law enforcement can address the gap in having suitable local experts to determine appropriate care. South Dakota sheriff departments got an opportunity to use technology with the VCC pilot program to virtually bring a behavioral health professional to individuals in crisis. This mixed methods study examined VCC encounter characteristics and perceptions by rural law enforcement users.

VCC consultations were frequently used for males and individuals with suicidal ideation or depression. These findings are similar to the results of existing studies on different crisis response models [7,12], although requests for aggressive or disruptive behaviors in crisis responses occurred infrequently during the VCC pilot program. This result shows the importance of having a behavioral health professional address the issue of potential self-harm and recommend proper care that is potentially lifesaving [6,9]. By having the behavioral health professional's evaluation and recommendation for next-step care, law enforcement saved time and resources previously expended to conduct IVCs [10]. Moreover, this type of program provides an opportunity to connect individuals in mental health crises with appropriate care and resources and to avert their involvement in the criminal justice system [31,32].

The potential mitigation of IVCs was seen as 1 of VCC’s primary benefits conveyed by law enforcement during qualitative interviews. Other benefits include access to behavioral health professionals virtually because those experts are rarely available locally in rural areas. This virtual access led to crisis situations being diffused, safety plans being initiated, and better documentation of care plans and rationales. Otherwise, the limited resources and expertise in rural settings often serve as significant hurdles to providing appropriate care for individuals with mental health issues [20,33,34]. The use of technology and the availability of virtual experts can potentially overcome multiple hurdles, as shown in this study. However, technology-related challenges (ie, technical concerns and network availability) should be anticipated and addressed before program implementation.

Insights from the interviews showed that introducing technology and relevant collaborative training in the implementation phase is crucial to building law enforcement confidence and familiarity with the program. This finding is consistent with the implementation of a coresponder program in an urban area, where initial agency collaboration and team building were identified as key facilitators of the program [35]. Moreover, it is essential for law enforcement to understand their jurisdiction characteristics and resource limitations to foster motivation and buy-in for program implementation. Indeed, 1 barrier previously identified for crisis response program effectiveness is a lack of buy-in and support from related agencies [10,20]. Future implementation efforts should consider team-building activities and identify key values of the service in the local context to facilitate successful preparation.

Our study also gathered insights into service use patterns following the VCC preparation. The process of presenting the VCC service, providing space for the virtual consultation, and making a decision based on expert recommendations via a structured protocol can be considered for future use. Indeed, systematic studies have highlighted the importance of building a standardized practice for replicability and evaluation purposes without neglecting the local context [5,9]. The availability of virtual experts does not diminish law enforcement’s role in this situation. This study shows that law enforcement officers are still the primary contact and decision makers. They provided the service with a proactive approach, while considering individuals' safety. The VCC program and technology provided law enforcement with tools to make informed decisions and facilitated better care for needed individuals. This social partnership model is perceived positively not only by law enforcement but also by individuals in crisis [36].

A prominent model for implementation in public service sectors has outlined 4 implementation phases (ie, exploration, preparation, implementation, and sustainment), including the influencing factors [37]. This study provides informative insights into law enforcement’s experience during the early phases of VCC implementation. Future studies can build upon the findings of this study and develop a focus on ways to make this crisis response model sustainable and analyze its cost-effectiveness.

### Limitations

There are a couple of limitations to this study. First, the interviewees were limited to those who participated in the VCC program and research and reasons for not participating were not captured. However, the quantitative data covered a diverse group of jurisdictions and the qualitative data provided rich insights into the VCC implementation experience. Moreover, the current results are similar to the existing literature in terms of the characteristics of the persons served, while extending the application of the coresponder program model into rural settings using virtual technology. Second, for outcome disposition, there were several encounters for which no final disposition could be ascertained from counties. As a result, the true proportion of concordance between recommended and actual dispositions may differ. Third, we do not have information about the counterfactual condition, meaning that we cannot yet compare the effectiveness of the VCC program with counties that do not have access to the program or the condition before the implementation of the program. This limitation can be the next direction of future relevant projects.

### Conclusion

Per the VCC pilot program operating in South Dakota, we found evidence from both quantitative and qualitative data that using telehealth to engage behavioral health professionals in mental health crisis encounters may positively impact efforts in addressing mental health crises. It has potentially saved individuals the stress and negative health outcomes associated with IVCs, it helps law enforcement by better preparing them to serve their local communities, and it helps counties preserve resources and reallocate time and energy otherwise dedicated toward IVCs.

Noting the acceptance and benefit to rural counties, Avera eCARE reached out to South Dakota legislators to promote the service statewide to all 66 counties. In the 2021 session, the South Dakota legislature supported the continuation and expansion of VCC services in the state [38]. As of this writing, VCC has been implemented in and serves 40 counties in South Dakota [39].

## Abbreviations

CIT: crisis intervention team
IVC: involuntary committal
VCC: virtual crisis care
